# Insanity in Child-Bearing

**Published:** 1887-05-14

**Authors:** Henry Rayner

**Affiliations:** Medical Superintendent, Hanwell Asylum


### Insanity in Child-Bearing.

By Henry Rayner, M.D., Medical Superintendent, Hanwell Asylum.

A Lecture delivered at the Midwives' Institute and Trained Nurses' Club.

(Continued from p. 90, vol. ii.)

### II.—Other Forms of Mental Disorders.

**The Mode of Onset of Mental Disorder.**

The form of the mental disorder was markedly related to the period of onset. Mania predominated at the earlier dates; melancholia and stupor at the later. The mode of onset of the mental disorder was most important and interesting, since the early detection of the oncoming mental distress might possibly prevent infanticide or suicide. We must next describe the sudden change which comes over the mother, and touch in detail on the initial symptoms under the heads of mania, melancholia, and stuporous insanity. Of mania there are two types—a febrile or delirious and a non-febrile or neurotic. The febrile type is by much the most rapid in its onset, active mental disorder being established in a few hours. The bodily symptoms of this distressful mental state are restlessness, sleeplessness, headache, vertigo, loss of appetite, etc. The non-febrile or neurotic types of mania again differ from the febrile form in the onset being more gradual, in the incoherence and excitement being less active, and in the mental disorders being more systematised. The hereditary tendency is usually strongly developed in these cases. The febrile type not infrequently resulted fatally at an early date, usually within the first two months, and occasionally much earlier in cases which were due to blood-poisoning, and might almost be regarded as puerperal fever, with maniacal delirium. If the patient escaped a fatal issue, the prospect of recovery was very good. The mania in some instances passed into a stuporous condition, and at other times into melancholia. The non-febrile or neurotic cases of mania commonly kept their character through the whole course of the disorder, and were usually more prolonged and less curable.

**Melancholia.**

Melancholia was far less common than mania. Melancholic cases were apt to prove recurrent in after pregnancies, and were less hopeful in regard to recovery. The stuporous form of insanity was rarely primary, and usually followed a more or less prolonged attack of melancholia or mania.

**Dangers to be guarded against.**

In all these forms of mental disorder there are three things to be guarded against, each requiring special attention on the part of those in charge, viz., the danger of infanticide, of suicide, and the refusal of food. The safety of the infant was the first thing to be considered. The child should be at once removed. The hatred or aversion which the insane mother evinced towards the infant was also sometimes extended to the husband and other near relatives, who should also keep away from the patient. The suicidal impulse demanded serious attention. In the febrile delirious form all attempts at suicide were usually impulsive and unpremeditated, being suggested by opportunities which presented themselves. In the non-febrile cases and melancholics the acts were much more deliberate, premeditated, and persistent. In all forms the necessity for food was so great that, where there was any refusal, the patient should be fed by force, if it could not be administered by tact and management. We will now give a few hints on the subject of feeding. We found, in the earlier years of our practice, that some attendants were eminently successful, while others were the reverse. We accordingly set ourselves to discover wherein the difference lay. We found that the successful attendants were those who diverted the attention of the patient. All had, of course, heard of grooms hissing when they were rubbing down their horses, and many may have thought, perhaps, that it was silly. It was, however, founded on a physiological basis. The groom was diverting the attention of the horse between its senses of feeling and hearing. So, too, the successful attendants divided the attention of their patients between their sense of sight, of feeling, and of hearing, while the unsuccessful ones did not. By adopting this as a basis of teaching we have for years past practically banished the stomach-pump and other implements of forcible feeding.

**Recovery.**

In regard to the results of these forms of insanity, recovery, when it occurred, was usually gradual and steady, relapses being very rare. The mental improvement was also preceded and accompanied by a great improvement in the bodily health, and in the weight of the body. The apparently most severe cases recovered in the largest proportion, the milder ones being less curable. Death, if it occurred, usually happened within the first two months. In an ordinary delivery the loss of bodily weight was equal to one-fifth of the total weight of the body, and in cases of insanity it was more. The scales, therefore, played an important part in measuring progress. The treatment, in fact, in uncomplicated cases was directed solely to getting the patient stout as soon as possible, and the most liberal diet should be employed.

**Transitory Insanity of the Parturient State.**

We will next say a few words in regard to the transitory forms of insanity, which occurred during the parturient state. It lasted only a few hours, or at most a day or so. They were probably familiar with the difficulty which occurred in the later stages of labour from the loss of all self-control by the patient; how some individuals lost all courage, and became abject in their fear, while others became desperate and almost frantic. This uncontrolled desperation might rise to a blind unreasoning fury, ending, perhaps, in infanticide or even suicide. The transitory mania had also been described as occurring within the first two or three days after delivery. The patients, after these attacks, sometimes had no recollection of what had passed, and in other cases the recollection was like that of a dream. These rare forms of mental disorder were of importance, chiefly from their bearing on medico-legal questions in cases of infanticide.

**Insanity during Gestation.** In regard to the forms of insanity which occurred during gestation, these were much less frequent than in the puerperal state, it being described as a cause in only .7 per cent. of the admissions to English asylums. From the statistics, it also appeared to be much less common among the rich than among the poor, a fact due perhaps to the greater efforts made by the former to avoid asylum treatment.

**Lactational Insanity.** Lactational insanity was a form applied to cases occurring during nursing, and not earlier than six to eight weeks after delivery. Of all female cases admitted to asylums, rather more than two per cent. were ascribed to this cause, so that it was about a third less frequent than puerperal insanity, and three times more frequent than the insanity of pregnancy. It was essentially a disease of the poorer classes, being at least three times more numerous in pauper than in private asylums. The reason of this is obvious, when we recognise the fact that these disorders were especially due to exhaustion, induced by prolonged suckling, hard work, anxiety, privation, and the unhealthy conditions to which the poor were generally exposed. There were very few fatal cases of lactational insanity. The great object in treatment should be to improve the nutrition, and the dietary of the patient should be of the most liberal description, with tonics and plenty of light and air.

**The Demeanour of Nurses to Insane Patients.** In conclusion, a few words in regard to the proper demeanour of nurses to an insane person may usefully be spoken. It should be calm, quiet, and controlled, remembering that in the raving of delirium the patient still retained some appreciation of her surroundings, and formed her judgments—extravagant though they might be—of the persons she saw. Quiet, firm persistence would enable nurses to do what was right, without provoking violent resistance, which brusque, harsh, or inconsiderate treatment was certain to produce. They should avoid deceptions and arguments in regard to delusions, a proceeding which would only tend to fix them more firmly in the mind; but, on the contrary, they should never acquiesce in the truth of these delusions. If they were founded on hallucinations (on false impressions made on the senses), it was well, either directly or indirectly, to show the error of them, where they could be proved to be fallacious, as in the supposed death of the husband, children, etc., who could be produced. And nurses should never forget, however apparently wilful might be the conduct of the patient, that it was insane wilfulness, which, if it succeeded in producing annoyance or irritation, would be sure to be repeated. In fact, in dealing with these cases a nurse needed an unlimited supply of the patience and sympathy which she cultivated in the ordinary discharge of her duties.

